# Polyethylene glycol-coated collagen patch (hemopatch^®^) in open partial nephrectomy

**DOI:** 10.1007/s00345-021-03827-x

**Published:** 2021-09-03

**Authors:** Michael Staehler, S. Rodler, M. Schott, J. Casuscelli, C. Stief, A. Spek, B. Schlenker

**Affiliations:** 1 Department of Urology, University Hospital Munich-Grosshadern, Ludwig Maximilian University, Munich, Germany; 2 Multidisciplinary Center On Renal Tumors, Department of Urology, University of Munich, Marchioninistr. 15, 81377 Munich, Germany

**Keywords:** Renal cell carcinoma, Open partial nephrectomy, Blood loss, Hemopatch, Sealant, Hemostasis

## Abstract

**Purpose:**

To describe the results of a polyethylene glycol-coated collagen patch, Hemopatch^®^ on blood loss, surgical time and renal function in partial nephrectomy (PN) for renal cell carcinoma (RCC).

**Methods:**

Out of a single surgeon cohort of *n* = 565 patients undergoing conventional open PN (CPN) between 01/2015 and 12/2017 at the University of Munich a consecutive subgroup (*n* = 42) was operated on using a polyethylene glycol-coated collagen-based sealant Hemopatch^®^ (Baxter International Inc., Deerfield, IL, USA) (HPN).

**Results:**

Median age was 65.2 years (range 12.7–95.2) with median follow-up of 9.43 months (0.03–49.15). Baseline renal function (CKD-EPI) was 78.56 ml/min/1.73 m^2^ (range 20.38–143.09) with a non-significant decline to 74.78 ml/min/1.73 m^2^ (range 3.75–167.74) at follow-up.

In CPN 46% had low complexity, 33% moderate complexity and 20% high complexity lesions with 33% low, 40% moderate and 27% high complexity masses in HPN.

Median tumor size was 4.3 cm (range 1–38 cm) in CPN with 4.8 cm (range 3.8–18.3 cm) with HPN, *p* = 0.293. Median blood loss and duration of surgery was significantly lower in the HPN group vs. CPN (146 ml ± 195 vs. 114 ml ± 159 ml; *p* = 0.021; 43 min ± 27 for HPN vs. 53 min ± 49; *p* = 0.035) with no difference in clamping time (12.6 min ± 8.6 for HPN vs. 12.0 min ± 9.5; *p* = 0.701).

**Conclusions:**

Hemopatch^®^ supported renoraphy shows promising results compared to standard renoraphy in PN. No side effects were seen. Further studies should evaluate the prevention of arterio-venous or urinary fistulas. In complex partial nephrectomies Hemopatch^®^ supported renoraphy should be considered.

## Introduction

Partial nephrectomy (PN) has become the standard of care in localized renal cell carcinoma (RCC) [1–3]. With oncologic equivalence the benefits include a reduced risk of renal insufficiency and prolonged survival. Although overall survival was not superior in a prospective randomized trial, the magnitude of data hint towards a prolonged overall survival with the preservation of renal parenchyma [3–5]. Challenges in PN are warm ischemia, tumor control, urinary fistulas and bleeding. The complexity of a renal lesions can predict the complications and challenges of the lesion as described with the R.E.N.A.L. score and other scoring systems [6, 7]. The main problem in renal surgery remains the control of bleeding, especially with larger and more complex lesions. The standard surgical technique to achieve that is suturing and bringing parenchyma on parenchyma with a renoraphy. Evidence exists that the use of a the sealing patch Tachosil^®^ (Ethicon Inc., Sommerville, NJ, USA) can reduce the duration of bleeding, but evidence is lacking that the blood loss can be reduced by hemostatic agents [7]. After initial reports on the successful use of Hemopatch^®^ (Baxter International Inc., Deerfield, IL, USA), a polyethylene glycol-coated collagen patch, [8–10] we intended to analyze its impact on surgical parameters in open PN.

## Methods

We report on a consecutive single surgeon cohort of patients who underwent a partial nephrectomy between 01/2015 and 12/2017. *N* = 564 patients undergoing open partial nephrectomy were identified within a prospective institutional database at the Department of Urology, University of Munich. All patients were operated using the same standardized surgical approach.

A consecutive subgroup of the last series of patients (*n* = 42) were operated on using a polyethylene glycol-coated collagen-based sealant Hemopatch^®^ (Baxter International Inc., Deerfield, IL, USA). All patients had an open midline approach and no further selection criteria have been used, other than indication for open partial nephrectomy.

### Surgical technique

Open PN was carried out following the same principle in a standardized approach. After identification and preparation of the kidney the renal hilum is identified and tunneled with a Guyon clamp to grasp a vessel-loop (Vesseloops™ Maxi, Medica Europe BV, NL) that is bolted around itself and the renal vessels (renal artery and vein). To open the sling easily, a ligature is inserted into the vessel-loop sling.

Perirenal fat is then removed completely from the kidney with a bipolar scissor sparing the fat on top of the tumor. The renal capsule is incised with an electrocautery needle or a scalpel followed by resection of the tumor with small Reynolds’ scissor. We do not enucleate but resect tumors avoiding the tumor pseudo capsule and leaving a margin of normal parenchyma covering the tumor. To achieve a complete resection in larger tumors the fat surrounding the collecting system is identified and the resection-line follows this fat at the ground of the tumor. The resected part of the kidney is controlled optically to ensure complete resection and tumor ground biopsies as well as the whole tumor are sent for immediate frozen cross section.

Larger vessels are controlled during the resection with cross-stitches using a small half round needle (RB1) and Prolene or Monocryl 4-0 RB1 (Ethicon Inc., Sommerville, NJ).

If necessary, the collecting system is closed with a running suture using Monocryl 4-0 RB1. Remaining vessels are identified and controlled either with a small needle (RB1) or a larger needle (SH) with PDS or Monocryl 4-0. While closing the parenchymal vessels the sutures are placed in a way to primarily reconstruct the kidney and approximate the parenchyma. To close the renal incision a running double-layered cross-stitched suture using Maxon 3-0 and a V20 needle (Covedien Syneture, Mansfield, MA) is performed according to the principles of Donati, modified into a running suture back and forth. If any, further bleeding is controlled with Maxon 3-0 cross-stitches.

Resection of the tumor is performed off clamp until bleeding of more than 200 ml occurs or visibility to identify the resection borders safely is impaired. If so, the vessel-loop is pulled to clamp the renal hilum. With an expected clamping time of more than 17 min, crushed ice is placed around the kidney immediately after clamping. Unclamping is performed by pulling the ligature inserted into the vessel loop.

### Hemopatch

Hemopatch^®^ is a polyethylene glycol-coated collagen patch. We used a standard size of 45 × 45 mm. To activate the coating and achieve bleeding control, slight pressure with a dry gauze for 2 min is recommended. There are three different ways to apply Hemopatch to the renal resection site. One is to place it into the resected area prior to a renoraphy and have it covered by the parenchyma. The rational for this procedure is to prevent urinary and arterio-venous fistulas. Another option is to place it over the resection wound and then place sutures through it to close the resection cavity and achieve a renoraphy. This could be an option especially in patients with limited time to perform the renoraphy as ischemia time is an issue, e.g., single renal units, or in complex lesions with an expected longer duration of reconstructive renoraphy. The third option is to just apply the Hemopatch to the wound cavity and not support it with sutures, which would be used in smaller, peripheral resections or lacerations of the renal capsule only.

### Administration of Hemopatch

**Fig. 1:**
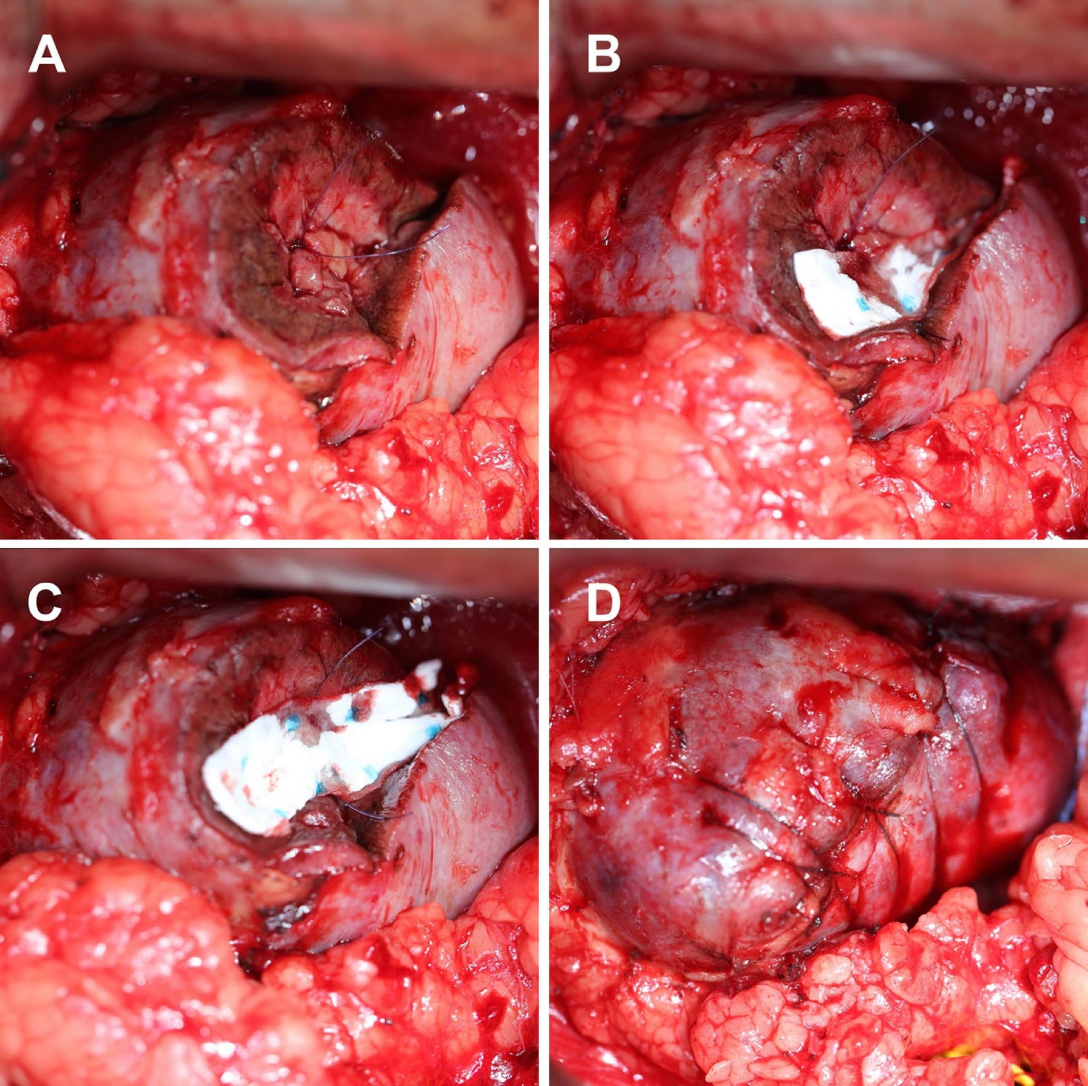
Renoraphy over Hemopatch; larger vessels and the collecting system are closed (**A**) initially before Hemopatch is placed (**B**) to prevent bleeding and arterio-venous fistula. In larger resections, a second layer of Hemopatch might be placed (**C**) before reconstruction of the renal parenchyma using a running suture (**D**)

**Fig. 2:**
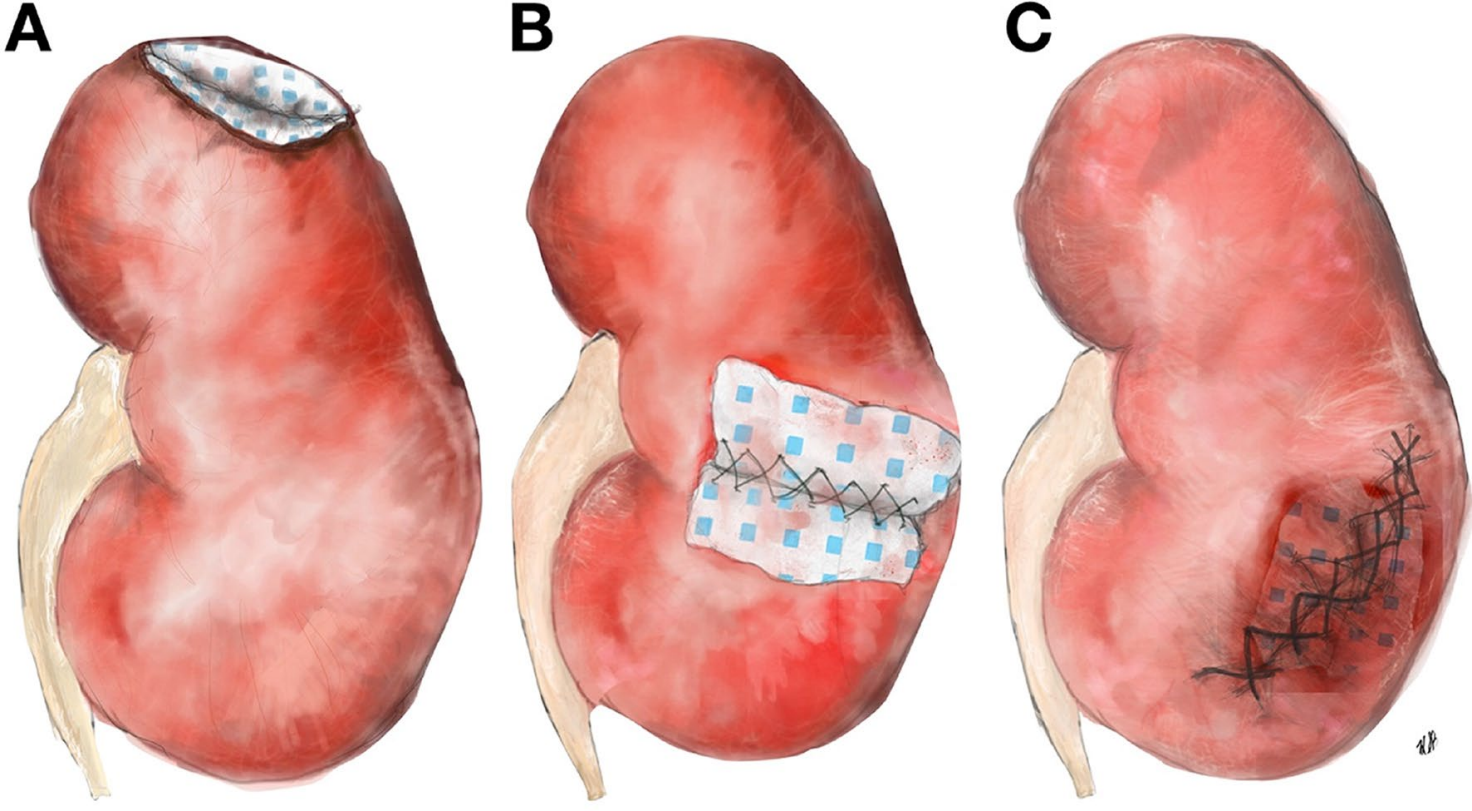
usage of Hemopatch in OPN; **A** Hemopatch covering resected tumor bed without sutures; **B** renoraphy over Hemopatch patch embedded into the resection wound **C** renoraphy with intraparenchymtous Hemopatch and primary closure (illustrations by K. Battle)

Hemopatch is used in different ways. Either in whole to cover the resection bed. This can be done with or without renoraphy, with sutures running through the Hemopatch. Or the Hemopatch is cut in smaller patches of 1 × 4.5 cm and placed into the resection bed, prior to the renoraphy (see Figs. 1, 2).

### Statistical analysis

The difference between HON and CPN results was assessed with the Mann–Whitney *U* test. Changes in laboratory values from baseline were compared between treatments with the Mann–Whitney *U* test. The differences between treatments were tested by a two-sided Fisher exact test. Calculations were done using IBM SPSS statistics version 25.

## Results

Median age was 65.2 years (range 12.7–95.2). The majority of the patients was male (67.8%). A6 patients had a single renal unit and 51.5% of the tumors were right sided. Median follow-up was 9.43 months (0.03–49.15). Baseline renal function as measured by CKD-EPI was 78.56 ml/min/1.73 m^2^ (range 20.38–143.09) with a non-significant decline to 74.78 ml/min/1.73 m^2^ (range 3.75–167.74) at follow-up.

*N* = 524 underwent conventional partial nephrectomy without Hemopatch supported renoraphy (CPN) and 42 patients had a PN with Hemopatch support (HPN).

R.E.N.A.L. Score was available for *n* = 324 patients in the CPN cohort and all HPN patients. In CPN 46% had a low complexity mass, 33% a moderate complexity and 20% a high complexity lesion. In HPN 33% ha a low, 40% a moderate and 27% a high complexity mass. Oncological results are not reported here but there were no positive margins at final pathology in CPN and HPN.

Median tumor size was 4.3 cm (range 1–38 cm) in CPN as compared to 4.8 cm (range 3.8–18.3 cm) with HPN, *p* = 0.293. The median blood loss and duration of surgery was significantly lower in the HPN group vs. CPN (114 ml ± 159 vs. 146 ml ± 195 ml; *p* = 0.021; 43 min ± 27 for HPN vs. 53 min ± 49; *p* = 0.035) with a clamping time that did not differ between the two groups (12.6 min ± 8.6 for HPN vs. 12.0 min ± 9.5; *p* = 0.701). The median duration of surgery also was significantly shorter in HPN. Median creatinine at discharge was similar in both groups (see Table 1).

**Table 1:** Comparative results of patients undergoing Hemopatch vs non-Hemopatch supported renoraphy; *: significant difference

	Non-hemopatch *N* = 524	Hemopatch *N* = 42	*p*
Age (years)	63.5 ± 13.6	64.2 ± 11.3	0.293
Tumor size cm	4.3 ± 2.6	4.8 ± 2.9	0.293
R.E.N.A.L. score low Intermediate High	46% 33% 20%	33% 40% 27%	– – –
Clamping time min	12.0 ± 9.5	12.6 ± 8.6	0.701
Creatinine at discharge mg/dl	1.3 ± 0.7	1.1 ± 0.3	0.012*
Duration of surgery min	53 ± 49	43 ± 27	0.035*
Blood loss ml	146 ± 195	114 ± 159	0.021*

## Discussion

According to the guidelines for the treatment of RCC, PN is the standard of care for the treatment of localized renal masses suspicious for RCC [11, 12]. The challenges of this procedure remain oncological equivalence to radical nephrectomy, bleeding, urinary fistulas and arterio-venous malformations [3, 13–15]. Several surgical approaches to the kidney are established, including laparoscopy and robot assisted laparoscopy. So far it cannot be determined, which surgical approach is the best for the preservation of renal function or the reduction of intra- and postoperative complications and if there are oncological differences between these procedures.

We report on a large series of patients treated with open PN by a single surgeon to eliminate the effects of expertise and a learning curve. We compare the intra-operative outcome of the renoraphy performed with or without the use of a NHS-PEG-coated, absorbable collagen-based sealant to provide hemostasis (Hemopatch^®^). This sealant has shown to be a valuable tool in the control of bleeding without any adverse events being reported so far across various surgical specialties [8]. Some evidence exists that Hemopatch^®^ can be used in urological procedures, predominantly renal surgery [9].

As proposed, hemostatic sealants are thought to prevent urinary fiistulas [16]. In our series we cannot comment on that, as we did not see any fistulas with our technique, but it could be speculated that in line with the results of others, these can be avoided. Therefore, a larger series of patients would be needed, as the rate of urinary leaks is usually below 2%. Besides urinary leaks we found that the creatine at discharge was significantly lower in HPN, but this might be a selection bias, so that we are not sure, that we can conclude that Hemopatch^®^ can preserve the renal function. Besides, this encouraging finding urges for a larger trial.

We could demonstrate that the administration of Hemopatch^®^ is safe and does not cause additional complications or side effects. Furthermore, the blood loss and duration of surgery in our series could be significantly lowered, even in a surgical setting, were bleeding is generally well controlled. What has not been estimated, was the duration until cessation of bleeding, which could be included in further studies. But with our setup of a late unclamping technique bleeding usually is controlled after unclamping, so that only in the minority of cases the duration of bleeding would be accessible. The problem with all hemostatic sealants remains, that their effect on the surgical outcome can be hardly assessed, as the result of any surgical procedure is, amongst others, a control of bleeding by the end of the procedure. Time to control mainly depends on the coagulation parameters of the blood and the patience and skills of the performing surgeon, thus the estimated impact of sealants will remain unclear.

In our series, we show that even in higher complexity lesions Hemopatch^®^ is a valuable tool to reduce the blood loss and thus is not only helping the patient to have a shorter surgical time and better post-operative outcome, but also gives the surgeon the comfort of a more reliable bleeding control. Blood loss itself might be correlated to a worse oncological outcome. It has been shown, that the perioperative transfusion of blood is related to a worse oncological outcome (HR 1.79) [18]. Thus, any effort must be taken to reduce the loss of blood during and after surgery to avoid transfusions.

Hemostatic agents so far have not shown to reduce the blood loss, but the time to bleeding control. Mainly TachoSil^®^ has proven to effectively reduce the bleeding time in OPN as shown by Siemer et al. [7]. In a series analyzing the use of Tachosil or Flowseal no significant improvements versus no hemostatic sealants in blood loss was seen but the hemostatic sealants seem to have prevented arterio-venous fistulas. The sample size thus was too small to show statistical significance differences between the groups [17]. A French group also was not able to show a benefit of the use of hemostatic agents in robotic partial nephrectomy [18]. Using the autologous fibrin glue Vivostat^®^ in 10 patients in laparoscopic partial nephrectomy immediate hemostasis could be achieved and have been confirmed by another group in 28 patients again. But there was no comparison or further evaluation of the patient cohorts [19, 20].

Although the reduction of median blood loss in the hemostatic sealant cohort does not warrant or allow further clinical conclusions, this still is an unexpected finding, as one would estimate that the bleeding always will be controlled at the end of the surgical procedure. But it offers comfort to the surgeon knowing that it is safe to resect even more complex lesions and still have excellent bleeding control. We therefore propose the use of Hemopatch^®^ in complex partial nephrectomies.

## Conclusions

Hemopatch supported renoraphy shows promising results compared to standard renoraphy in PN. No side effects were seen. Further studies should evaluate the prevention of arterio-venous or urinary fistulas. In complex partial nephrectomies Hemopatch^®^ supported renoraphy should be considered.

## References

[1] Ljungberg B, Albiges L, Abu-Ghanem Y, Bensalah K, Dabestani S, Fernandez-Pello S (2019). European association of urology guidelines on renal cell carcinoma: the 2019 update. Eur Urol.

[2] Van Poppel H, Da Pozzo L, Albrecht W, Matveev V, Bono A, Borkowski A, Colombel M, Klotz L, Skinner E, Keane T, Marreaud S, Collette S, Sylvester R (2011). A prospective, randomised EORTC intergroup phase 3 study comparing the oncologic outcome of elective nephron-sparing surgery and radical nephrectomy for low-stage renal cell carcinoma. Eur Urol.

[3] Weight CJ, Lieser G, Larson BT, Gao T, Lane BR, Campbell SC, Gill IS, Novick AC, Fergany AF (2010). Partial nephrectomy is associated with improved overall survival compared to radical nephrectomy in patients with unanticipated benign renal tumours. Eur Urol.

[4] Dabestani S, Beisland C, Stewart GD, Bensalah K, Gudmundsson E, Lam TB, Gietzmann W, Zakikhani P, Marconi L, Fernandez-Pello S, Monagas S, Williams SP, Torbrand C, Powles T, Van Werkhoven E, Meijer R, Volpe A, Staehler M, Ljungberg B, Bex A (2018). Long-term outcomes of follow-up for initially localised clear cell renal cell carcinoma: RECUR database analysis. Eur Urol Focus.

[5] Ljungberg B, Hedin O, Lundstam S, Warnolf A, Mandahl Forsberg A, Hjelle KM, Stief CG, Borlinghaus C, Beisland C, Staehler M (2016). Nephron sparing surgery associated with better survival than radical nephrectomy in patients treated for unforeseen benign renal tumors. Urology.

[6] Kutikov A, Uzzo RG (2009). The R.E.N.A.L. nephrometry score: a comprehensive standardized system for quantitating renal tumor size, location and depth. J Urol.

[7] Chang X, Liu T, Zhang F, Qian C, Ji C, Zhao X, Liu G, Guo H (2015). The comparison of R.E.N.A.L., PADUA and centrality index score in predicting perioperative outcomes and complications after laparoscopic radio frequency ablation of renal tumors. J Urol.

[8] Siemer S, Lahme S, Altziebler S, Machtens S, Strohmaier W, Wechsel HW, Goebell P, Schmeller N, Oberneder R, Stolzenburg JU, Becker H, Luftenegger W, Tetens V, Van Poppel H (2007). Efficacy and safety of TachoSil as haemostatic treatment versus standard suturing in kidney tumour resection: a randomised prospective study. Eur Urol.

[9] Lewis KM, Ikeme S, Olubunmi T, Kuntze CE (2018). Clinical effectiveness and versatility of a sealing hemostatic patch (HEMOPATCH) in multiple surgical specialties. Expert Rev Med Devices.

[10] Imkamp F, Tolkach Y, Wolters M, Jutzi S, Kramer M, Herrmann T (2015). Initial experiences with the Hemopatch(R) as a hemostatic agent in zero-ischemia partial nephrectomy. World J Urol.

[11] Fingerhut A, Uranues S, Ettorre GM, Felli E, Colasanti M, Scerrino G, Melfa GI, Raspanti C, Gulotta G, Meyer A, Oberhoffer M, Schmoeckel M, Weltert LP, Vignolini G, Salvi M, Masieri L, Vittori G, Siena G, Minervini A, Serni S, Carini M (2014). European initial hands-on experience with HEMOPATCH, a novel sealing hemostatic patch: application in general, gastrointestinal, biliopancreatic, cardiac, and urologic surgery. Surg Technol Int.

[12] Ljungberg B, Bensalah K, Canfield S, Dabestani S, Hofmann F, Hora M, Kuczyk MA, Lam T, Marconi L, Merseburger AS, Mulders P, Powles T, Staehler M, Volpe A, Bex A (2015). EAU Guidelines on renal cell carcinoma: 2014 update. Eur Urol.

[13] Escudier B, Porta C, Schmidinger M, Algaba F, Patard JJ, Khoo V, Eisen T, Horwich A, Group EGW (2014). Renal cell carcinoma: ESMO Clinical Practice Guidelines for diagnosis, treatment and follow-up. Ann Oncol.

[14] Lee DJ, Hruby G, Benson MC, McKiernan JM (2010). Renal function and oncologic outcomes in nephron sparing surgery for renal masses in solitary kidneys. World J Urol.

[15] Thompson RH, Lane BR, Lohse CM, Leibovich BC, Fergany A, Frank I, Gill IS, Blute ML, Campbell SC (2010). Every minute counts when the renal hilum is clamped during partial nephrectomy. Eur Urol.

[16] Riggs SB, Larochelle JC, Belldegrun AS (2008). Partial nephrectomy: a contemporary review regarding outcomes and different techniques. Cancer J.

[17] Rane A, Rimington PD, Heyns CF, van der Merwe A, Smit S, Anderson C (2010). Evaluation of a hemostatic sponge (TachoSil) for sealing of the renal collecting system in a porcine laparoscopic partial nephrectomy survival model. J Endourol.

[18] Soria F, de Martino M, Leitner CV, Moschini M, Shariat SF, Klatte T (2017). Perioperative allogenic blood transfusion in renal cell carcinoma: risk factors and effect on long-term outcomes. Clin Genitourin Cancer.

[19] Tonyali S, Koni A, Yazici S, Bilen CY (2017). The safety and efficacy of adjuvant hemostatic agents during laparoscopic nephron-sparing surgery: comparison of tachosil and floseal versus no hemostatic agents. Urol J.

[20] Peyronnet B, Oger E, Khene Z, Verhoest G, Mathieu R, Roumiguie M, Beauval JB, Pradere B, Masson-Lecomte A, Vaessen C, Baumert H, Bernhard JC, Doumerc N, Droupy S, Bruyere F, De La Taille A, Roupret M, Bensalah K (2015). The use of hemostatic agents does not prevent hemorrhagic complications of robotic partial nephrectomy. World J Urol.

